# Eine frühe Dissertation zur Endourologie – Leben und Werk von Georg Adelmann (1811–1888)

**DOI:** 10.1007/s00120-025-02653-y

**Published:** 2025-08-22

**Authors:** Friedrich H. Moll, Erki Tammiksaar, Giacomo Padrini, Thorsten Halling, Nils Hansson

**Affiliations:** 1 Institut für Geschichte, Theorie und Ethik der Medizin, Universität Düsseldorf, Düsseldorf, Deutschland; 2 Curator Museum, Bibliothek und Archiv zur Geschichte der Urologie, Deutsche Gesellschaft für Urologie e. V., Düsseldorf Berlin, Düsseldorf, Deutschland; 3 Urologische Klinik, Kliniken der Stadt Köln GmbH, Neufelder Straße 32, 51067 Köln, Deutschland; 4 Centre for Health and Society, Düsseldorf, Deutschland; 5 Centre of Science Studies Karl Ernst von Baer House, University of Life Sciences, Tartu, Tartu, Estland

**Keywords:** Promotion, Dissertationsforschung, Akademische Qualifikationsarbeiten, Hochschulrecht, PhD, MD thesis, Dissertation research, PhD thesis, Qualification in medicine, Constitution of universities

## Abstract

Medizinische Dissertationen ermöglichen präzise Einsichten in den Wissenstand und in die Forschungsmethodik zu einer bestimmten Zeitepoche. Im Gegensatz zu eher kanonisierten Lehrbuchtexten werden in Dissertationen wissenschaftliche Diskurse und unterschiedliche Auffassungen oftmals kleinschrittiger und damit nachvollziehbarer dargestellt. Damit sind sie einschlägige Quellen, um die Entwicklung eines Spezialgebiets nachzuvollziehen und zu analysieren, sind jedoch häufig nicht, auch aufgrund unzureichender Dokumentation, im Fokus der wissenschaftshistorischen Forschung. Im Kontext eines Forschungsprojekts zur Dissertationskultur in der Urologie wird in diesem Beitrag am Beispiel des Dorpater Chirurgen Georg Adelmann die Einbettung einer frühen Dissertation zur Endourologie in die zeitgenössische Forschungsdiskussion untersucht.

## Abstract (en)

Doctoral dissertations in medicine provide precise insights into the state of knowledge and research methodology at a particular time. In contrast to more canonised textbook texts, dissertations often present scientific discourses and different views in a more detailed and therefore more comprehensible manner. This makes them relevant sources for tracing and analysing the development of the specialist field, but they are often not a focus of research into the history of science, partly due to insufficient documentation. In the context of a research project on dissertation culture in urology, this article uses the example of the Dorpat surgeon Georg Adelmann to analyse the embedding of an early dissertation on endourology in the contemporary research debate.

## Zur Einleitung

Dissertationen werden in der Medizin viel stärker als in den meisten anderen Fakultäten als ein integraler Bestandteil der universitären Ausbildung wahrgenommen. Im Wettbewerb um den qualifizierten Nachwuchs für das eigene Fachgebiet werben auch einige urologische Universitätskliniken durchaus engagiert um Doktoranden.^1^ Auch medial sehr präsent ist die hochschulpolitische Auseinandersetzung um die Organisation der Promotion in der Medizin, insbesondere in Hinblick auf die Anforderungen [1] und Qualitätssicherung [2].

In historischer Perspektive werden Dissertationen als einschlägige Quellen zur Analyse der Wissens- und Wissenschaftsentwicklung der Medizin herangezogen [3]. Zu den Untersuchungsgegenständen gehören die Promovenden selbst und ihre Betreuer [4], der Sichtbarkeit von Dissertationen [5], u. a. auch die Bedeutung der jeweiligen Wissenschaftssprache [6]. Einen besonderen Stellenwert nehmen Dissertationen in der fachkulturellen Erinnerung der jeweiligen medizinischen Disziplinen ein. In der Urologiegeschichte wurde beispielsweise an die bahnbrechende Dissertation *Étude sur le Rein des Urinaires* von Joaquin Albarran (1860–1912) [7], die Bedeutung von Etienne-Frédéric Bouisson (1813–1884) für die chirurgische Behandlung der Hypospadie [8] oder die erste promovierte Urologin Helena Maria Kornella (1897–1992) in Polen erinnert [9].

Neue Impulse sind von dem aktuellen Trend zur Digitalisierung bislang kaum zugänglicher Dissertationssammlungen in Archiven und Bibliotheken und den damit verbundenen neuen Möglichkeiten serieller Textanalysen zu erwarten [10, 11].

Im Kontext eines Forschungsprojekts zur Dissertationskultur in der Urologie [12] wird in diesem Beitrag am Beispiel des Dorpater Chirurgen Georg Adelmann die Einbettung einer frühen Dissertation zur Endourologie in die zeitgenössische Forschungsdiskussion untersucht. Auf Grundlage von publizierten und archivalischen Quellen, insbesondere für seine Zeit an der Universität Dorpat (heute Tartu, Estland), soll die Bedeutung dieser Qualifikationsarbeit im Gesamtwirken dieses Mediziners herausgearbeitet werden.

Im national überhöhten Sprachduktus der Zeit heißt es im Jahr 1900 in der *Allgemeine Deutsche Biographie*:

„Georg Franz Blasius Adelmann gehört zu den hervorragenderen Gelehrten deutschen Ursprungs, welche auf russischen Boden verpflanzt, die deutsche Wissenschaft dort zur Blüthe brachten und so wesentlich zur culturellen Hebung Rußlands auf die moderne Stufe beitrugen“ [13].

## Eine Karriere von Marburg nach Dorpat

**Abb. 1:**
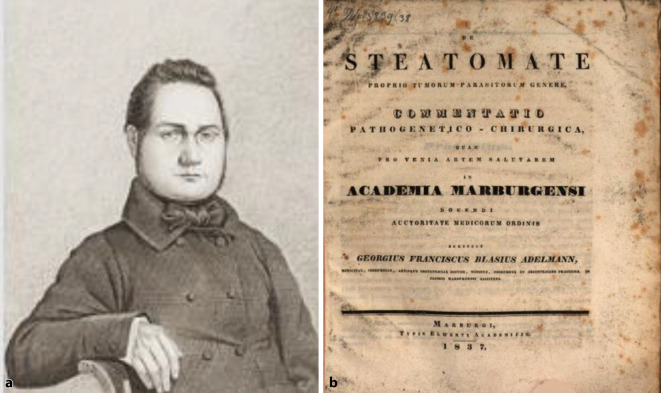
**a** Georg F. B. Adelmann (1811–1888; Federzeichnung 25, 5 × 30 cm, beschnitten). In: Ferdinand Justi, Icones Professorum Marpurgensium. Museum für Kunst und Kulturgeschichte der Philipps-Universität Marburg (Inventar-Nr. 28.229) Hessische Biographie online: https://www.lagis-hessen.de/de/subjects/rsrec/sn/bio/register/person/entry/adelmann%252C+georg%252A+franz+blasius%20online. (Reproduktion Moll-Keyn, mit freundl. Genehmigung). **b** Habilitationsschrift aus Marburg 1837 (Reproduktion Moll-Keyn, mit freundl. Genehmigung)

Georg Franz Blasius Adelmann wurde am 28. Juni 1811 als erster Sohn von Vinzenz Ferrerius Adelmann (1780–1850) und seiner Ehefrau Theresia Eva Vincentia (1780–1818), Tochter eines Würzburger Stadtrates, geboren. Sein Vater wirkte als Arzt am Fuldaer Landkrankenhaus Wilhelmshospital, dessen Bruder Franz Joseph Adelmann (1787–1868) wiederum war Lehrstuhlinhaber für Naturwissenschaften an der Universität Leuven. Hier studierte Georg Adelmann zunächst Naturwissenschaften zwischen 1825–1828, nachdem er in Würzburg das Gymnasium besucht hatte. Es folgte 1828–1831 ein Medizinstudium an der Philipps-Universität Marburg, das er an der Julius-Maximilians-Universität Würzburg fortsetzte. Er wurde im August 1832 in Würzburg promoviert und legte 1833 das medizinische Staatsexamen ab.^2^ Ab 1833 war er dann „Gehilfsarzt“ an der Medizinischen Abteilung des Krankenhauses in Marburg, 1834 Reskript als Arzt, Wundarzt und Geburtshelfer mit dem Wohnsitz in Fulda. Am dortigen Wilhelmhospital vertrat er den Chirurgen und Geburtshelfer^3^ [14]. 1835 legte er die Staatsprüfung als Gerichtsarzt ab, war aber ab 1837 als „Gehilfsarzt“ am chirurgischen Klinikum in Marburg unter Christoph Ullmann (1773–1849) wieder akademisch tätig. Hier habilitierte er sich am 2. Dezember 1837 mit der Schrift „De steatomate“ [15] und wurde dort Privatdozent^4^ (Abb. 1). In dieser Eigenschaft hielt er Vorlesungen über Chirurgie einschließlich Augenheilkunde, Bandagenlehre, „Materia medica“ und „Rezeptierkunst“.

**Abb. 2:**
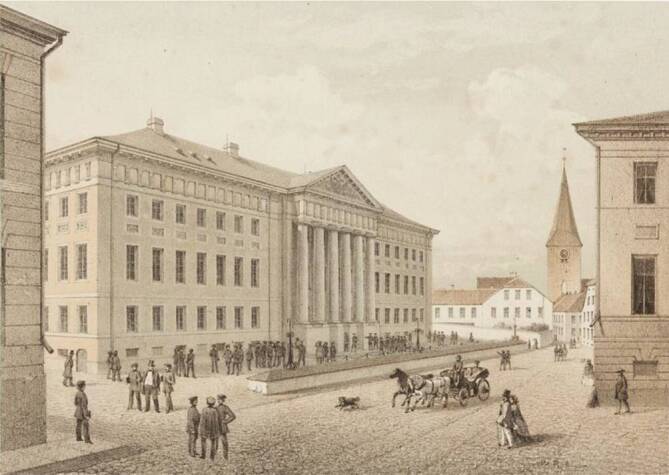
Hauptgebäude der Universität 1860 Album von Dorpat und Umgebungen, Verlag der Lithographischen Anstalt von L Höflinger, Dorpat. Wikicommons

Im Juli 1841 wurde er als Nachfolger von Nikolai Iwanowitsch Pirogow (1810–1881) auf den Lehrstuhl für Chirurgie und Augenheilkunde an die kaiserliche Universität Dorpat (heute Tartu ülikool) berufen ([16, 17]; Abb. 2). Sein Gegenkandidat war der spätere Professor für Chirurgie an der Universität Marburg Eduard Zeis (1807–1868).^5^ Das Universitätskonsil hatte am 17. Januar 1841 Adelmann zum Professor gewählt und die staatliche kaiserliche Bestätigung hierzu am 4. März 1841 erhalten.^6^

**Abb. 3:**
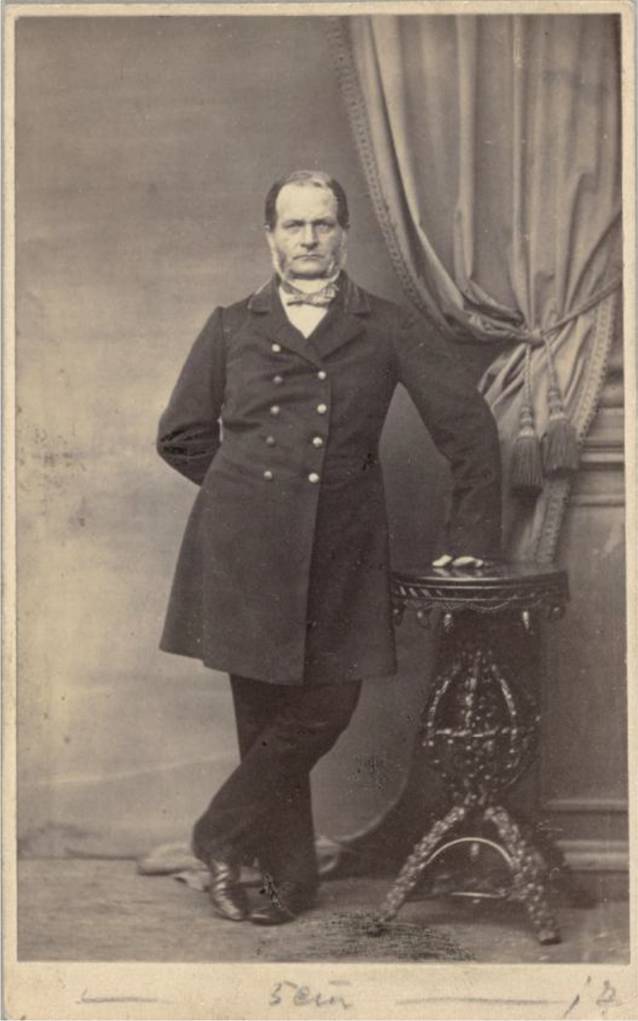
Georg Adelmann, wahrscheinlich um 1860, in typischer Position auf einen Tisch gestützt aufgrund langer Belichtungszeiten von Studio-Portraitaufnahmen (Universitätsarchiv Tartu/Estnisches Nationalarchiv AM_13741_131F17380_1). (Repro Erki Tammiksaar, mit freundl. Genehmigung)

Kurz vor seiner Übersiedlung nach Dorpat heiratete Georg Adelmann im Februar 1841 Amalie Maria Barkhausen (1816–1868), Tochter eines Rentmeisters aus Marburg. Adelmann sollte 25 Jahre in Dorpat lehren, was damals für einen Universitätsprofessor üblich war, um die volle Pension zu erhalten^7^ (Abb. 3). Im Jahre 1871 wurde er emeritiert und zog nach Berlin.

Auch hier war er noch fachpolitisch tätig und eröffnete 1882 die Gründungssitzung der Berliner chirurgischen Gesellschaft [18]. Adelmann starb in Berlin im Jahr 1888 [19, 20].

Im Nekrolog der Dorpater Tageszeitung *Neue Dörptsche Zeitung* wurde die Bedeutung von Adelmann für Dorpat folgendermaßen von den Zeitgenossen gewürdigt:

„Im Jahre 1841 trat er seine Lehrtätigkeit an der hiesigen Hochschule an und ein volles Menschenalter hat er als akademischer Lehrer und Operateur unter uns erfolgreich gewirkt, dabei durch mehrfache Abhandlungen auf dem Gebiete der plastischen Chirurgie und Knochen-Resectionen seine Wissenschaft fördernd. Der Nachfolger Pirogow’s und der Vorgänger und Lehrer unseres jetzt so berühmten Landsmannes Dr. Ernst von Bergmann, hat er sich um die Disciplin der Chirurgie an unserer Hochschule vielfache Verdienste erworben, insbesondere auch eine ansehnliche Zahle tüchtiger Operateure herangebildet. Als Lehrer der studierenden Jugend wie als Mensch hat er sich in weiten Kreisen allgemeiner Achtung zu erfreuen gehabt: seine Ehrenhaftigkeit, sein offenes, gerades Wesen, seine Liebenswürdigkeit erwarben ihm überall Freunde und an Beweisen der Anerkennung hat es ihm nicht gefehlt“ [21].

In einem weiteren Nachruf in der *St. Petersburger medicinische Wochenschrift*, der offensichtlich von seinem Schüler Eduard von Wahl (1833–1890), Professor für Chirurgie an der Universität Dorpat und Redakteur der Zeitschrift, stammt, wurde betont, dass Adelmann „eine grosse Anzahl tüchtiger Schüler herangebildet“ habe [22].

Als seine Schüler sind besonders Georg von Oettingen (1824–1916) und Eduard von Wahl (beide später in Dorpat als Professoren tätig), Christian von Hübbenet (1822–1873), Julius von Szymanowski (1829–1868) und Theodor Bornhaupt (1842–1905; alle drei an der Universität Kiew) und Wilhelm Grube (1827–1898) und Carl von Reyher (1846–1890; beide an Universität von Charkow tätig) hervorzuheben [23].

**Abb. 4:**
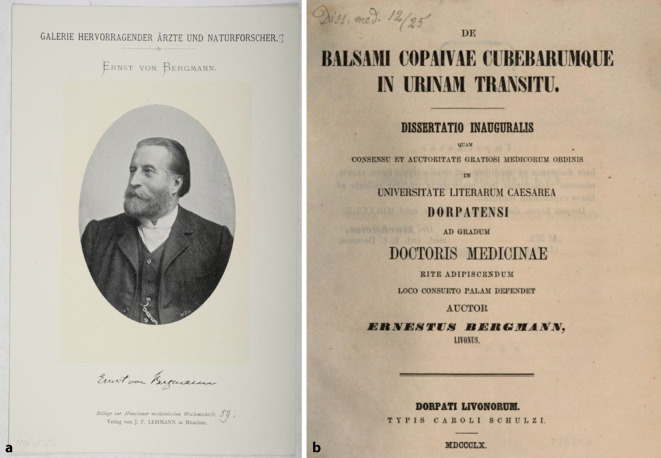
**a** Ernst von Bergmann (1836–1907). Lehmanns Galerie hervorragender Ärzte und Naturforscher. Lichtdruck 1796 (Repro Moll-Keyn, Sammlung Moll). **b** Dissertation Ernst von Bergmann Würzburg 1860. (Repro Moll Keyn, mit freundl. Genehmigung.) Obwohl ein Zeitunterschied von fast 30 Jahren besteht, ist der formale Aufbau von Dissertationen auch noch in der Mitte des 19. Jahrhunderts analog dem Jahrhundertbeginn. Latein ist noch Wissenschaftssprache für diese traditionelle Publikations- und Qualifizierungsform in einer Hochschulschrift. Exemplar der Bayrischen Staatsbibliothek. (Repro Moll-Keyn, mit freundl. Genehmigung)

Sein Schwiegersohn und Nachfolger im Amt, Ernst von Bergmann (1836–1907)^8^, in Riga geboren, wurde ebenfalls mit einer frühen urologischen Arbeit über die Ausscheidung des Kopaivabalsams^9^ und von Kubebarum (De balsami Copaivae Cubebarumque in urinam transitu; [24]) in Dorpat 1860 promoviert (Abb. 4). Mit seinem Namen sind eine Form des Flankenschnittes („nach von Bergmann/Israel“; [25]) und eine Technik der Hydrozelenoperation („nach von Bergmann/Winckelmann“) verknüpft [26]. Von Bergmanns akademische Karriere führte von Dorpat über Würzburg nach Berlin, ein möglicher Hinweis auf die Netzwerke des Schwiegervaters.

### Adelmanns wissenschaftliches Werk und seine zeitgenössische Anerkennung

Georg Adelmann wirkte in der Zeit zwischen Einführung der Allgemeinanästhesie Mitte des 19. Jahrhunderts [27] und Einführung der Listerschen Antisepsis zu Beginn der 1870er-Jahre [28] im deutschsprachigen Raum. Daher lagen seine Schwerpunkte in Dorpat bei Eingriffen im Extraperitonealraum wie dem Hals-Nasen-Raum oder an den Extremitäten [29].

**Abb. 5:**
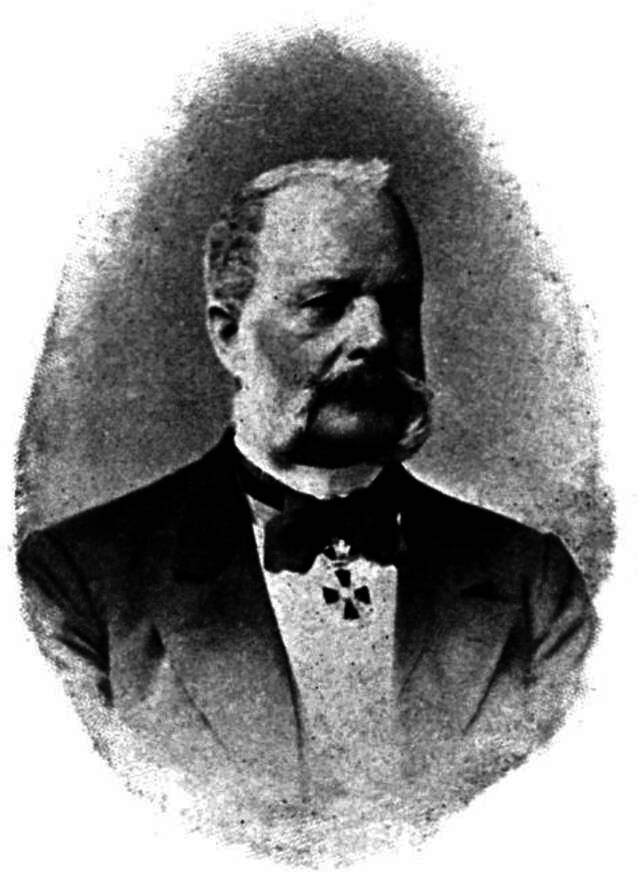
Georg von Adelmann (1811–1888) aus Pagel J (1901). Biographisches Lexikon hervorragender Ärzte des 19. Jahrhunderts. Urban und Schwarzenberg, Berlin – Wien Sp. 10–12. (Repro Moll-Keyn, mit freundl. Genehmigung)

Das Eponym „Adelmann-Operation“ ist in der chirurgischen Literatur zur Bezeichnung einer Operation zur Handverschmälerung noch immer gebräuchlich.^10^ Seine wissenschaftlichen Arbeiten umfassen generalistisch die technischen Möglichkeiten der operativen Medizin/Chirurgie zu diesem Zeitpunkt, wobei detailliert auch über 122 urologische Krankheitsfälle aus Dorpat zumeist „Blenorrhö“ 54 und „Hydrozelen“ 18 Fälle kursorisch publiziert wurden, wobei 53 Operationen (von 471 insgesamt) vorgenommen wurden [30]. In die gesamte Publikation floss auch eine Beschreibung der Lebensumstände der Bevölkerung im Sinne einer medizinischen Topographie, die zu dieser Zeit ein beliebtes Literaturgenre war, mit ein ([31, 32]; Abb. 5).

**Abb. 6:**
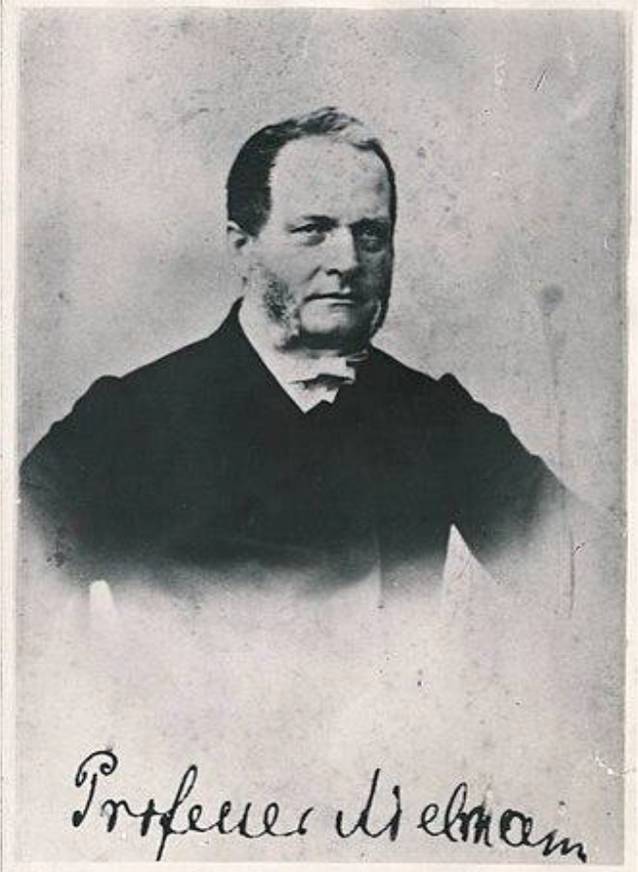
Georg von Adelmann (TM F 569:36) Tartu Linnamuuseum; TMF569_36_1_pisipil online: https://www.muis.ee/museaalView/3191010. (Repro Moll-Keyn, mit freundl. Genehmigung)

Bereits im Jahre 1855 wurde Georg Adelmann in die Deutsche Akademie der Naturforscher Leopoldina (Nr. 1727)^11^ gewählt, 1849 zum Staatsrat und 1860 zum wirklichen russischen Staatsrat erhoben^12^, was auch sein Gehalt vergrößerte. Die besondere Wertschätzung für sein Wirken als Chirurg und Leiter der chirurgischen Klinik wurde von Seiten der Universität mehrmals gewürdigt. Der Zar ließ ihm persönlich sogar die von Summe von 429 Rubel in Silber zukommen^13^ und erhielt im Jahr 1855 den Russischen Stanislausorden II. Klasse und den St. Annen Orden II. Klasse im Jahre 1869.^14^ Seit 1866 war er ebenfalls Träger des Belgischen „Membre étranger honoraire“^15^ (Abb. 6).

Im Jahr 1900, also lediglich 12 Jahre nach seinem Tod, wurde im Beitrag zu Adelmann in der Allgemeine Deutsche Biographie betont:

„Als fleißiger Arbeiter war A. beinahe ein halbes Jahrhundert lang litterarisch thätig. Seine namhaftesten Schriften sind: ‚De Dignitate Lithontritiae‘, 1833 (Diss.), ‚De steatomate‘, 1837 (Habilitationsschrift), ‚Untersuchungen bei krankhaften Zuständen der Oberkieferhöhle‘, Dorpat 1844, mit 3 Tafeln“ [33].

Von Adelmanns zeitgenössischer Anerkennung soll nun am Beispiel seiner Dissertation eine retrospektive Einordnung seines wissenschaftlichen Werks gegenübergestellt werden.

## Adelmanns Dissertation (1832) im Kontext der Harn- und Blasensteintherapie zu Beginn des 19. Jahrhunderts

**Abb. 7:**
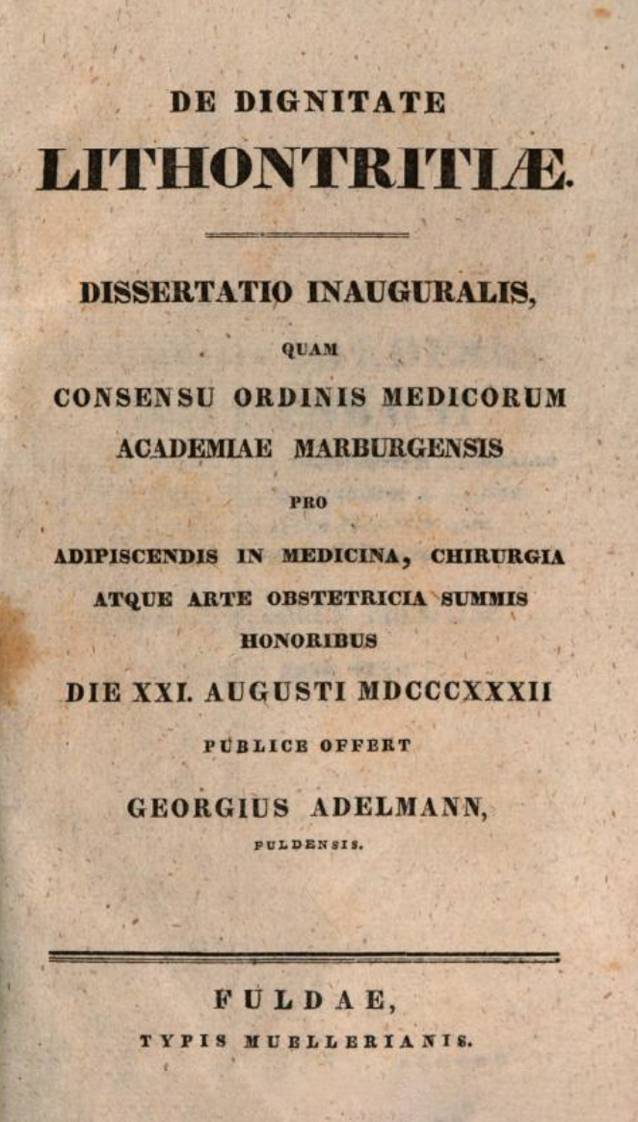
Georg Adelmann 1832 De Dignitate Lithontritiae, Frontispiz. (Repro Moll-Keyn, mit freundl. Genehmigung)

Nach Abschluss seines Studiums in Marburg legte Adelmann im Jahre 1832 seine Dissertation „De Dignitate Lithontritiae“ der Universität Würzburg vor, die in Fulda gedruckt wurde, (Abb. 7).^16^

Adelmanns Thema aus dem Bereich der Harn- und Blasensteintherapie ist zu diesem Zeitpunkt eines von vielen Beispielen für die Verwissenschaftlichung der Medizin. Spätestens zu Beginn des 19. Jahrhunderts war die Standardisierung des offenen Steinschnitts, in der Regel der Seitensteinschnitt („Sectio lateralis“), seltener die „Sectio alta“ in der deutschsprachigen operativen Medizin standardisiert [34, 35], der Übergang von handwerklich ausgebildeten Operateuren zu akademisch ausgebildeten Ärzten abgeschlossen und im staatlich organisierten Regelwerk zur Berufsausübung fest verankert. Zeitgleich entwickelte sich von Paris ausgehend das erste minimalinvasive Operationsverfahren in Urologie und Medizin durch Jean Civiale (1792–1867), klinisch ab 1824 verbreitet. Diese Technik löste sofort einen heftigen innerfachlichen Diskurs aus [36–43].

Adelmann wurde, wie er im Vorwort dankend erwähnt, durch seinen akademischen Lehrer Cajetan Textor (1782–1860) zu dem Thema angeregt. Textor, Ordinarius für Chirurgie in Würzburg und Oberwundarzt am dortigen Juliusspital, führte selber die offene Lithotomie und ab 1827 in Würzburg auch die Civialsche Lithotripsie durch [44, 45].

Klassisch beginnt Adelmann in § 1 Introductio mit einer historischen Herleitung aufgrund der besonderen Bedeutung des Harnsteinleidens:

„Morbum calculi antiquissimis jam temporibus medicorum mentem occupasse, multa remedia testantur, quae jam eo tempore sub nomine remediorum lithontripticorum laudebantur …“ [Die Steinkrankheit beschäftigte die Ärzte schon in der Antike, wie viele Heilmittel belegen, die bereits damals unter dem Namen Lithontriptika gepriesen wurden.]

Hierbei bezieht er sich auf klassische Texte wie Hippokrates und Celsus. Er zitiert originale Quellen sowie eine Dissertation von Hartenkeil (1785) aus Würzburg [46]. Danach geht er auf die Wortschöpfung aus dem Griechischen ein [47]. Bis heute hat sich gerade bei der Harnsteintherapie eine regelmäßige historische Herleitung im urologischen Schrifttum erhalten, was die Fachkonstitution des Querschnittfaches durch eine historische Methodologie unterstreicht [48–51].^17^

**Abb. 8:**
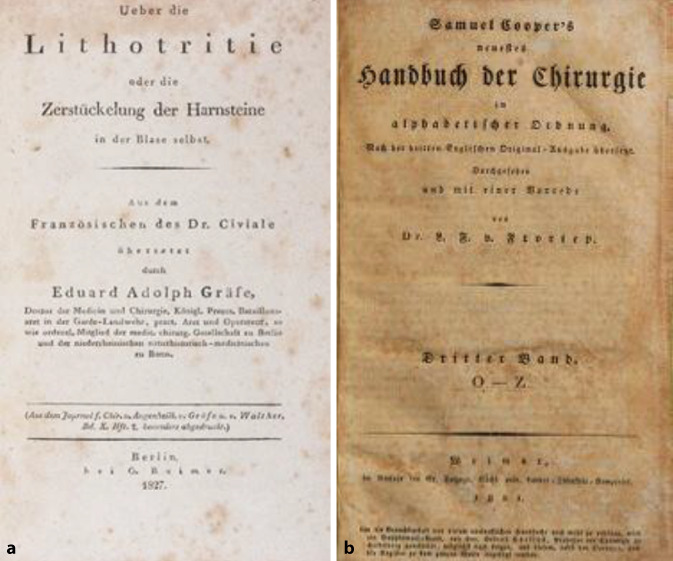
**a** Eduard Adolph von Gaefe (1794–1859) 1827 über die Lithotritie oder die Zerstückelung der Harnsteine in der Blase selbst. Sonderdruck aus *Journal der Augenheilkunde*, Reimer, Berlin. (Repro Moll-Keyn, mit freundl. Genehmigung). **b** Samuel Cooper (1780–1848) 1821 deutschsprachige Ausgabe seines Wörterbuches bei Reimer, Berlin „Neuestes Handbuch der Chirurgie in alphabetischer Reihenfolge“. (Repro Moll-Keyn, mit freundl. Genehmigung)

In § 2 führt Adelmann weitere operative Verfahren wie die Sectio alta und den Seitensteinschnitt an [52], wobei er zeitaktuelle Autoren wie von Kerns Lehrbuch 1828 [53] aus der deutschsprachigen Literatur zitiert. Ob er die weiteren Diskurschriften zur Methode von Civiale im Einzelnen kannte, kann anhand der Zitate nicht ermittelt werden. Von der englischsprachigen Literatur rezipiert er William Cheselden (1688–1752) über das zusammenfassende Werk von Samuel Cooper (1780–1848), das auch in deutscher Übersetzung 1821 erschienen war [54]. Arbeiten Civiales lagen Adelmann in einer deutschen Übersetzung durch den Berliner Chirurgen von Eduard Adolph von Graefe (1794–1859) aus dem Jahre 1827 vor (Abb. 8 und 9; [55]).

**Abb. 9:**
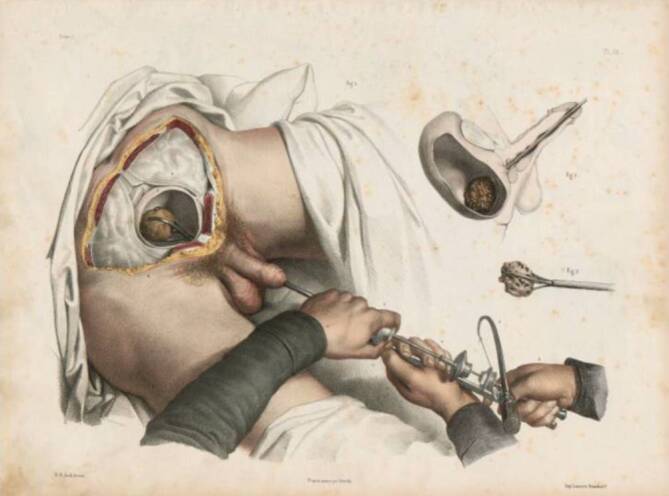
Bourgery, Jean Baptiste Marc (1797–1849) und Nicolas Henri Jacob (1782–1871) 1840 Traité complet de l’anatomie de l’homme: comprenant la médicine opératoire (Band 7, Atlas). Delaunay, Paris. Pl. 58. (Repro Moll-Keyn, mit freundl. Genehmigung)

**Abb. 10:**
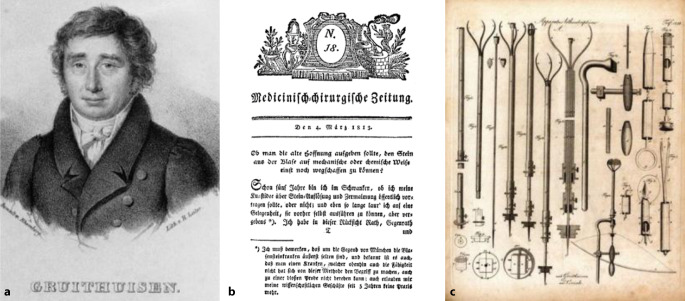
**a** Portrait Franz von Paula Gruithuisen (1748–1852), Stich; Altbestand DGU. (Repro Moll-Keyn, mit freundl. Genehmigung). **b** Frontispiz aus Nr. 18 Salzburger Medizinisch-Chirurgische Zeitschrift 1813; **c** Apparatus lithontripticus. Gruithisen’s Vorschlag und Civiales Verfahrensweise den Stein in der Blase zu zerbrechen. Froriep Chirurgische Kupfertafel Nr. 156. Hrsg. v. Ludwig Froriep (1779–1847), Heft Nr. 31, 1825, Weimar. (Repro Moll-Keyn, mit freundl. Genehmigung)

Bevor er sich der blinden Blasensteinlithotripsie in § 5 zuwendet, erwähnt er auch die von Franz von Paula Gruithausen (1747–1852) theoretisch angegebene „galvanische Methode“ [56–58]. Aus dieser frühen theoretischen Arbeit von 1813 hatte sich bereits innerhalb der französischen Urologie ein Prioritätsstreit um die Originalität der Civial’schen Methode entsponnen (Abb. 10).

**Abb. 11:**
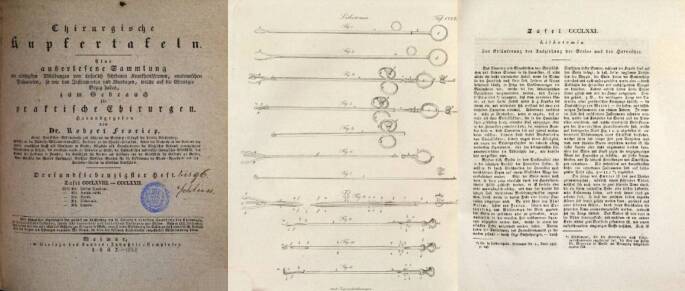
Robert Froriep (1804–1861) Chirurgische Kupfertafel Nr. 371, 1837, Weimar. (Repro Moll-Keyn, mit freundl. Genehmigung). Diese qualitativ hochwertigen Abbildungen standen Georg Adelmann zur Verfügung. Hier in einer späteren Ausgabe mit Instrumenten zur Entfernung von Konkrementresten aus der Harnröhre

In § 6 führt er 26 wichtige Arbeiten aus dem Zeitraum 1823–1828 zur Civiale-Methode an, um in einem weiteren Kapitel den Diskurs über die frühe Arbeit Gruithausens nicht unerwähnt zu lassen, wobei er Gruithausen das Primariat in der Frage zuschreibt „Gruithausen primus nostru …“ [59]. Ganz in der Gedankenwelt seines Lehrers verhaftet, der in Würzburg eine chirurgische Instrumentensammlung begründete, geht Adelmann zunächst auf die historischen Instrumente und ihre Autoren, wie auch in „Caput primum“ „Comperato instrumentorum lithontripticorum critica“ auf eine Auseinandersetzung mit den zeitgenössisch verwendeten Instrumente ein. Hierbei scheinen ihm (Frorieps) Chirurgische Kupfertafeln (1820–1846), die von Ludwig (1779–1847) später ab 1833 Robert Froriep (1804–1861) in einzelnen Heften in Weimar herausgegeben wurden und 1820 in vier Bänden erschienen, gute Dienste zu leisten, da diese mehrfach zitiert werden [60].^18^ Der Einführung bei Harnröhrenengen wird ein großes Gewicht beigemessen und die Brauchbarkeit der verschiedenen Instrumente ausführlich diskutiert (Abb. 11).

Ab § 20 wendet er sich der Operationstechnik zu, die er in kleine nachvollziehbare Teilschritte unterteilt: „Urethrae dilatatio“ „Situs aegri“, „Evacuatio intestini recti“, ab § 21 u. a. „Instrumenti immisio“, „Calculi tritura“, „Instrumenti extracto“.

In einem „Caput tertium“ setzt er sich ab § 22 mit den Argumenten der Gegner wie auch mit operativen Komplikationen wie „Debilitas vesicae“, „Febris“ und „De morbis renum“ oder „Apoplexia“ auseinander. In § 37 vergleicht er abschließend die neue Methode der Lithotripsie mit den offenen operativen Methoden, wobei er in der voränesthesiologischen Zeit besonders „Lithotritia minimus horribilis provocat, quam Cystostomia“, d. h. die deutliche Schmerzreduzierung hervorhebt ohne zu vernachlässigen, dass die technische Ausführung der Lithotripsie höhere Ansprüche an den Operateur stellt [61]. Ab § 38 führt Adelmann nochmals die Gegenanzeigen für die neue Operationstechnik an [62].

Auf S. 95 folgt noch ein Literaturnachtrag besonders französischer Autoren. Schon ganz dem naturwissenschaftlichen Prinzip der Zeit verhaftet, führt Adelmann mit Namensnennung von Patienten und Autoren eine Statistik von ausgeführten Operationen zwischen 1825–1827 an. Für uns heute unter Datenschutzgründen schwer vorstellbar, ermöglichte es die Namensnennung des Patienten einem Wissenschaftler selber nachzuforschen und sich auch später nach dem Wohlbefinden zu erkundigen.

## Zusammenfassung und Forschungsausblick

Georg von Adelmanns Dissertationsschrift *De Dignitate Lithontritiae* bildet retrospektiv betrachtet nicht nur den zeitgenössischen Forschungsstand adäquat ab, sondern ist in Umfang (120 Seiten) und formaler Gestaltung (u. a. vollständige Referenzen) späteren medizinischen Dissertationen weit voraus.

Der Beitrag zur Forschung kann nicht abschließend beurteilt werden, da sich die Einschätzungen in der biographischen Literatur, die Dissertation gehöre zu Adelmanns wichtigsten Werken durch weiterführende Anerkennungsnachweise, wie etwa Zitationen oder Rezensionen, bisher systematisch nicht haben verifizieren lassen.

**Abb. 12:**
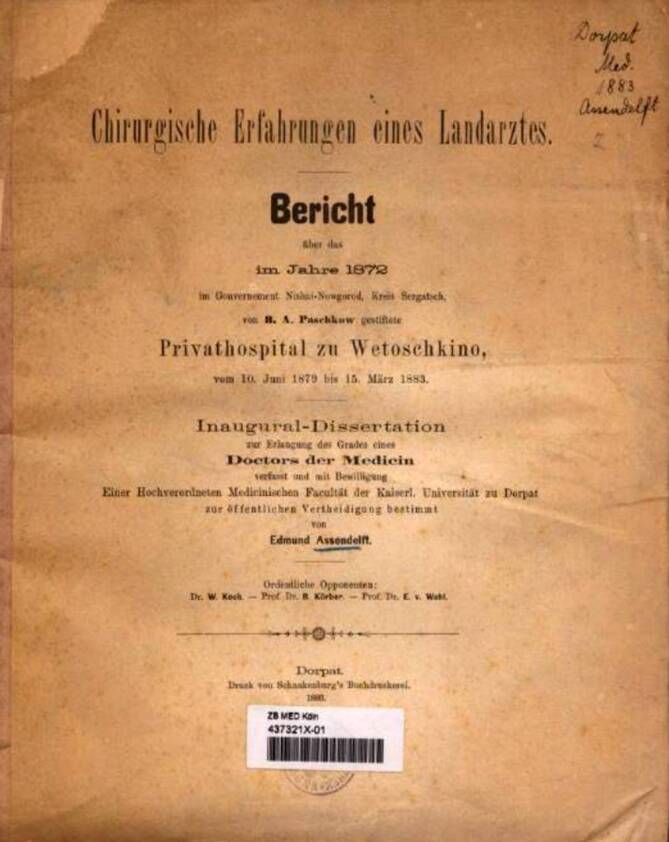
Dissertation aus Dorpat aus dem Jahre 1883 (ehemals aus dem Bestand der Universität Bonn, heute ZB Med Köln). (Online: https://digital.zbmed.de/chirurgie/content/pageview/9899910?query=lithiasis ZB Med Köln zugegriffen 12.06.2025)

Das zumindest die klinische Praxis auch ein halbes Jahrhundert später regional von der Operationstechnik nach Civiale abwich, die Adelmann so ausführlich beschrieben hatte, belegt ausgerechnet eine Dissertation aus Dorpat (Tartu) von 1883 (Abb. 12). Diese zeigt an Fällen eines russischen Krankenhauses aus den Jahren 1879–1883, dass die sehr technikaufwendige Lithotripsie eine lange Zeit gerade in ländlichen Gebieten benötigte, um sich durchzusetzen. Der Autor berichtet über operative Fälle eines kleinen russischen Krankenhauses. In diesem Zusammenhang wird die doch große Zahl von 41 Blasensteinen (bei 61 urologischen Fällen insgesamt) berichtet, die per „Sectio alta“ oder „Sectio lateralis“ operiert wurden. Während der Autor schulmäßig zu Fragen von Wunddrainage, Wundverschlusses, Krankenhausverweildauer, Patientenalter, usw. Stellung nimmt, wird die Technik der „blinden Lithotripsie“, die zum Zeitpunkt der Publikation bereits seit 60 Jahren eingeführt ist, bemerkenswerter Weise nicht diskutiert.

Eine Korrespondenz aus dem Jahr 1842 unterstützt die eingangs dieses Beitrags zitierte Einschätzung von einer Mittlerrolle Adelmanns zwischen deutscher und russischer Medizin. Auf die Anfrage der medizinischen Fakultät der Universität Dorpat zur Ausgestaltung eines medizinischen Journals, antwortet Adelmann begeistert, dass er bereits lange auf eine Zeitschrift gehofft habe, die zwischen deutscher und russischer Medizin vermitteln könne und die Dorpater Universität dafür ein geeigneter Standort sei. Er selbst habe geeignete Beiträge für ein solches Projekt bereitliegen. Adelmann erkundigt sich, ob deutsche Aufsätze gegen Abzug vom Honorar ins Russische übersetzt werden können. Falls ja, würde er die vorhandenen Manuskripte unverzüglich einsenden. Weiter fragt er an, ob auch ein Abdruck von Auszügen aus deutschen und russischen Arbeiten vorgesehen sei [63].

Dass Adelmanns Werk auch jenseits des Atlantiks wahrgenommen wurde, belegt eine ausführliche Besprechung einer klinischen Fallsammlung von 1879 [64] im *Boston Medical and Surgical Journal (*später *New England Journal of Medicine;* [65]).

Adelmann war als klinisch tätiger Chirurg sowie als Forscher ein Generalist seiner Zeit, so dass sein Dissertationsthema keine herausragende Bedeutung in seinem Gesamtwerk hatte.

## Fazit für die Praxis

- Medizinische Dissertationen ermöglichen, wie das Beispiel Adelmann zeigt, präzise Einsichten in den Wissenstand in die Forschungsmethodik zu einer bestimmten Zeitepoche. Im Gegensatz zu eher kanonisierten Lehrbuchtexten werden in Dissertationen wissenschaftliche Diskurse und unterschiedliche Auffassungen oftmals kleinschrittiger und damit nachvollziehbarer dargestellt.
- Weiterhin lassen sich akademische Höflichkeitsformen und wissenschaftliche Netzwerke der Lehrer wie auch der Schüler ablesen. Häufig bilden diese die Grundlage für weitere Forschungstätigkeiten im gleichen oder anderen Wissensbereichen.

## Footnotes

1 Die urologische Klinik der Universitätsmedizin Mannheim beispielsweise verweist unter dem Titel Promotion und Forschung in der Urologie auf eine „engmaschige Betreuung“ und 222 erfolgreich abgeschlossene Promotionen seit 1970, https://urologie-umm.de/doktoranden/ zugegriffen 24.06.2025

2 NN 1888 Nekrolog Adelmann Hessenland. *Zeitschrift für Hessische Geschichte und Literatur* 2 207 (02.07.1888) online: https://orka.bibliothek.uni-kassel.de/viewer/image/1289911336242_0002/215/

3 Baltische Historische Kommission 2025 *Adelmann Baltisches Biographisches Lexikon* online: https://bbld.de/0000000055335781 zugegriffen 15.05.2025

4 Georg Adelmann Landesgeschichtliches Informationssystem Hessen https://www.lagis-hessen.de/de/subjects/rsrec/sn/bio/register/person/entry/adelmann%252C+georg%252A+franz+blasius zugegriffen 15.05.2025

5 Medizinische Fakultät an das Conseil der Universität Dorpat, Dorpat, 7. December 1840 (Nationalarchiv Estlands, RA, EAA.402.3.10, Bl. 3–4v).

6 Curator des dorpatschen Lehrbezirks and das Conseil der Universität, Dorpat, 14. März 1841 (Nationalarchiv Estlands, RA, EAA.402.3.10, Bl. 12–12v).

7 Ministerium für Volksaufklärung an das Conseil der Universität, Riga, 18. October 1871 (Nationalarchiv Estlands, RA, EAA.402.3.10, Bl. 163).

8 Von Bergmann heiratete die Tochter Hildegard Luise Adelmann (1846–1868) im Jahre 1866

9 Innerlich bei Gonorrhö, Entzündung der Harnwege im 19. Jahrhundert angewendet

10 Operationsmethode zur Handverschmälerung durch Amputation bzw. Resektion eines Mittelhandstrahls inklusive Mittelhandköpfchen.

11 https://de.wikipedia.org/wiki/Liste_der_Mitglieder_der_Deutschen_Akademie_der_Naturforscher_Leopoldina/1855

12 Curator des dorpatschen Lehrbezirks an den Rektor der Universität, Dorpat, 8. März 1849 (Nationalarchiv Estlands, RA, EAA.402.3.10, Bl. 55–55v).

13 Curator des dorpatschen Lehrbezirks and das Conseil der Universität, Dorpat, 23. December 1849 (Nationalarchiv Estlands, RA, EAA.402.3.10, Bl. 62–62v).

14 Formulärliste über den Dienst, Ministerium für Volksaufklärung an den Rektor der Universität, Dorpat, 14. Februar 1869 (Nationalarchiv Estlands, RA, EAA.402.3.10, Bl. 142, 168v).

15 https://www.armb.be/l-academie/composition/armbmembrelist/detailmembre/?no_cache=1&tx_fwbarmb_fwbarmbfrt%5Bmembre%5D=554&tx_fwbarmb_fwbarmbfrt%5Baction%5D=show&tx_fwbarmb_fwbarmbfrt%5Bcontroller%5D=Membre&cHash=d1bb6ffbe5226ce2768d5b7a9ffdf337 zugegriffen 15.05.2025

16 Ein Digitalisat der Bayerischen Staatsbibliothek ist seit 2024 online verfügbar: https://www.digitale-sammlungen.de/de/view/bsb10847820?page=1

17 Antrittsvorlesung Universität Bonn Dr. med. Johannes Stein Vom Steinschneider zum Endourologen – Harnsteintherapie im Wandel der Zeit 20.06.2025 online: https://www.medfak.uni-bonn.de/de/fakultaet/veranstaltungen/antrittsvorlesungen/antrittsvorlesung-herr-dr-med-johannes-stein

18 „Die Abbildungen nach früheren und gleichzeitigen Autoren, meist schwarz-weiß, z. T. recht gut illuminiert, sich durchweg gut und sauber gestochen. Es sind die meisten klinischen und anatomischen Autoren der Zeit dort reproduziert, so daß man sich einen recht guten Überblich über die Kenntnisse und die Möglichkeiten verschaffen kann, der in den 30er-Jahren des 19. Jahrhunderts auf der Höhe von Wissen und Kunst seiner Zeit stand. Für unsere heutigen Gewohnheiten und Begriffe ist es auffällig, wie eingehend die einzelnen Operationsverfahren dargestellt und wie üppig die damaligen Operationsinstrumente ausgestattet waren. In dieser Hinsicht ist besonders zu erwähnen die Darstellung der Lithothripsie mit herrlichen Instrumenten, … So manche versteckt erschienen Abbildung, so manches entlegenes Werk ist durch diesen Sammelatlas auf die Nachwelt gekommen. …“ Goldschmidt E 1925 Entwicklung und Bibliographie der pathologisch-anatomischen Abbildung. Hiersemann, Leipzig S. 119–120
